# Editorial: Unraveling mechanisms and implications of anti-RBC antibodies in transfusion medicine

**DOI:** 10.3389/fimmu.2025.1671343

**Published:** 2025-08-13

**Authors:** Krystalyn E. Hudson, David R. Gibb

**Affiliations:** ^1^ Laboratory of Transfusion Biology, Department of Pathology and Cell Biology, Columbia University Irving Medical Center, New York, NY, United States; ^2^ Division of Transfusion Medicine, Department of Pathology and Laboratory Medicine, Cedars-Sinai Medical Center, Los Angeles, CA, United States

**Keywords:** RBC alloimmunization, anti-RBC antibodies, blood substitutes, CD47 antigen, antigen modulation, RBC clearance, antibody antigen interaction, antibody mediated enhancement

Red blood cells (RBCs) are indispensable for oxygen delivery and tissue perfusion, and their loss can result in life-threatening anemia. Antibodies against RBC antigens can be clinically significant through accelerated RBC clearance, hemolysis and antigen modulation; thus, they represent a critical intersection of immunology and clinical medicine with profound implications for transfusion support, transplantation, and pregnancy. Anti-RBC antibodies arise either as autoantibodies (i.e., autoimmune hemolytic anemia [AIHA]), or as alloantibodies, typically following exposure to non-self RBC antigens through transfusion, pregnancy, or transplantation (1). AIHA can be further characterized into warm and cold types, each with distinct immunopathologic mechanisms and clinical presentations (2). Alloantibodies, on the other hand, complicate transfusion compatibility and can lead to hemolytic transfusion reactions or hemolytic disease of the fetus and newborn (HDFN). The complexity of the human blood group system, with >360 antigens across 47 blood group systems (3), underpins the immunogenic potential of RBCs and the clinical challenges posed by anti-RBC antibodies.

Despite decades of clinical experience with anti-RBC antibodies and studies in animal models, key questions remain about how and why these antibodies form and best strategies to manage their consequences. This collection of manuscripts addresses these challenges from multiple angles by investigating the immunologic origins of antibody responses, effects of antibody:antigen interactions, innovative approaches to expand the blood supply, and new tools to better quantify the clinical burden of anemia. By bridging mechanistic insights with translational applications, these studies inform our scientific understanding and the practical care of patients affected by anti-RBC antibodies.

As RBC alloantibodies are formed in 3-5% of transfused patients in United States’ hospitals, there is great interest in identifying risk factors that contribute to RBC alloimmunization. Elucidating risk factors will allow for interventions, including extended antigen matching, for recipients with high risk. Recipient inflammation is one risk factor identified in both pre-clinical models and human studies (1). In this Research Topic, Paul et al. investigate the role of type 1 interferons in driving alloantibody production in lupus-prone mouse models. Their findings reveal that interferon signaling, not lupus-like pathology alone, is a key determinant of alloimmune responsiveness following RBC transfusion. Whether type 1 interferon activity, present in 40% of patients with lupus, contributes to alloimmunization in patients remains an unanswered question.

Once alloantibodies are produced, re-exposure to the RBC antigen during an incompatible transfusion can lead to enhanced clearance of antibody-bound RBCs. However, not all antibody:antigen interactions lead to clearance; antibodies can also modulate RBC antigen expression, thereby preventing clearance and improving RBC lifespan (4). RBC survival is also prolonged by RBC expression of CD47, which inhibits phagocytosis of RBCs (5). In this Research Topic, Jajosky et al. investigated the role of CD47 in regulating antibody-mediated RBC clearance versus antigen modulation. They observed that anti-RBC antibodies enhance clearance of CD47-deficient RBCs, indicating a novel interplay between CD47 and anti-RBC antibodies in RBC survival.

To further investigate the effects of antibody:antigen interactions, the same group examined RBC antibody binding kinetics. As RBC clearance and antigen modulation depend on Fc gamma receptors (FcγR) expressed by phagocytes (4), Jajosky et al. performed *in vivo* binding studies utilizing FcγR-deficient mice that expressed the Duffy RBC antigen. They found that antibody binding to the Duffy antigen at saturating levels was rapid, but dissociation was much slower. Interestingly, addition of cell-free antibody to antibody-bound RBCs led to binding of the free antibodies, suggesting that the antibody:antigen interaction is a highly dynamic process that has implications for RBC targeted therapies.

One such targeted therapy is passive infusion of anti-Rh(D) antibodies, which prevents Rh(D) alloimmunization and HDFN during pregnancy. However, anti-Rh(D) fails to prevent alloimmunization in a small percentage of pregnancies (6). One possible cause of ineffective anti-Rh(D) therapy comes from recent studies demonstrating that infusion of a specific IgG subtype, IgG2c, targeting an RBC antigen enhances, rather than inhibits, alloantibody responses (7). In this Research Topic, Qiu et al. report that the enhanced response follows binding of antigen-specific IgG2c to FcγRIV on dendritic cells. This finding indicates that antibody subtypes in anti-Rh(D) therapeutics may influence the efficacy or anti-Rh(D) during pregnancy.

Managing patients who have been sensitized to RBC antigens remains one of the most complex challenges in transfusion medicine, as finding compatible donor units becomes increasingly difficult. With blood donation rates declining globally, there is growing interest in alternative RBC sources, including *in vitro*-derived cells, universal donor engineering, and xenogeneic options. In this Research Topic, Park et al. investigated the compatibility of genetically modified porcine RBCs with human serum, demonstrating that targeted gene deletions significantly reduce complement activation, hemolysis, and phagocytosis, suggesting improved immunologic compatibility. Building on this work, Roh et al. further evaluated the safety and efficacy of transfusing triple-knockout porcine RBCs into nonhuman primates, showing transient hematologic benefit. While challenges remain, particularly regarding antibody formation and RBC clearance, these studies identify porcine RBCs as a potential alternative during blood shortages and a possible option for patients with complex alloantibody profiles.

Effectively managing patients with anti-RBC antibodies requires addressing the immunologic drivers of hemolysis and understanding and measuring the patient experience, particularly fatigue, which is among the most debilitating symptoms reported. While most relevant to AIHA, fatigue is also a common consequence of alloantibody-mediated hemolysis. In this Research Topic, Cella et al. validate the use of the FACIT-Fatigue scale in patients with cold agglutinin disease (CAD), demonstrating its reliability, responsiveness, and clinical relevance in capturing meaningful changes in fatigue. Complementary work in warm AIHA supports the scale’s content validity (8), suggesting its broader applicability across hemolytic conditions. Together, these findings provide a valuable tool for quantifying the patient reported outcomes and guiding therapeutic decision making in clinical trials and practice.

Together, these studies illuminate the multifaceted nature of anti-RBC antibodies, from the molecular mechanisms that govern their formation and function, to the clinical challenges they pose, and the innovative strategies being developed to address them. By integrating insights from basic science, translational models, and patient-centered outcomes, this body of work advances our understanding of both auto- and alloimmune responses to RBCs and has the potential to significantly improve the safety of RBC transfusions.
